# Performance comparison of three scaling algorithms in NMR-based metabolomics analysis

**DOI:** 10.1515/biol-2022-0556

**Published:** 2023-03-27

**Authors:** Xia Liu, Yiqun Fang, Haifeng Ma, Naixia Zhang, Ci Li

**Affiliations:** Department of Diving and Hyperbaric Medicine, Navy Medical Center, Naval Medical University (Second Military Medical University), Shanghai, 200433, China; Shanghai University of Sport, Shanghai 200438, China; CAS Key Laboratory of Receptor Research, Department of Analytical Chemistry, Shanghai Institute of Materia Medica, Chinese Academy of Sciences, Shanghai 201203, P. R. China

**Keywords:** metabolomics, nuclear magnetic resonance, CTR scaling, UV scaling, Par scaling, discriminative metabolites

## Abstract

Unit variance (UV) scaling, mean centering (CTR) scaling, and Pareto (Par) scaling are three commonly used algorithms in the preprocessing of metabolomics data. Based on our NMR-based metabolomics studies, we found that the clustering identification performances of these three scaling methods were dramatically different as tested by the spectra data of 48 young athletes’ urine samples, spleen tissue (from mice), serum (from mice), and cell (from *Staphylococcus aureus*) samples. Our data suggested that for the extraction of clustering information, UV scaling could serve as a robust approach for NMR metabolomics data for the identification of clustering analysis even with the existence of technical errors. However, for the purpose of discriminative metabolite identification, UV scaling, CTR scaling, and Par scaling could equally extract discriminative metabolites efficiently based on the coefficient values. Based on the data presented in this study, we propose an optimal working pipeline for the selection of scaling algorithms in NMR-based metabolomics analysis, which has the potential to serve as guidance for junior researchers working in the NMR-based metabolomics research field.

## 1 Introduction

The applications of metabonomics/metabolomics in multiple research fields have been booming in recent decades [1]. The large-scale, high-dimensional, and complicated metabolomics datasets grow markedly in number [2–4], which will be generally analyzed by multivariate analysis (MVA) approaches. Multivariate data mining tools, including principal component analysis (PCA), partial least squares discriminant analysis (PLS-DA), and orthogonal projections to latent structures discriminant analysis, are commonly used to provide scatter plots for illustrating the clustering and classification between or among metabolic profiles [5]. Undoubtedly, the key function of metabolic profiling analysis is to provide discriminable information between or among specimen groups [6,7]. Additionally, the clustering feature of an assumed sample group is also crucial for a better understanding of the metabolomics dataset [8]. The extraction of clustering information with high accuracy can improve the efficiency of data mining and minimize possible data misunderstandings [9]. Based on the known knowledge, the variation in obtained metabolomics results usually stems from the “technical” and/or “biological” variance among samples [10]. For NMR-based metabolomics studies, technical variance mainly derives from NMR data pretreatment processes [11], including the peak aligning operation and dataset scaling calculation. Proper NMR peak alignment and the precise selection of scaling methods are crucial for restricting the sample distinction to merely focus on biological variance arising from subtle differences in genetic, physiological, pathological, and/or environmental factors among individuals [12].

As mentioned above, the big data extracted from ^1^H NMR spectroscopy [13], liquid (or gas) chromatography-mass spectrometry [14], and/or other analytical techniques are routinely preprocessed to make them suitable for multivariate statistical analysis. For the pretreatment of the ^1^H-NMR dataset, the procedures are composed of several major steps, including phase and baseline correction, peak alignment, piecewise integration, normalization, and dataset scaling [15,16]. Unit variance (UV) scaling, centering (CTR) scaling, and Pareto (Par) scaling are three commonly used metabolomics dataset scaling algorithms. In the UV scaling treatment, the scaling weights of each data value are calculated as the inverse of standard deviation (1/stdev) [17,18]. CTR scaling converts the variance to fluctuate around the zero level, and the covariance of the variables is used for analysis [19,20]. For Par scaling, the mean-centered variables are divided by the square root of the standard deviation of each variable [20]. Interestingly, during our previous NMR-based metabolomics studies [21], we found that the performances of these three scaling methods were different from each other. We want to illustrate the performances of three scaling methods in NMR-based metabolomics analysis and set up guidance for the precise selection of scaling algorithms. In this study, we first investigated the dissimilarities of clustering patterns achieved by processing the same dataset with UV scaling, CTR scaling, Par scaling, and no scaling (NS) and then evaluated their performances in identifying the discriminative metabolites between the two groups. In comparison with CTR scaling, Par scaling, and NS, UV scaling presented a larger technical error tolerance when performing statistical analysis (PCA) by incorporating the human urine metabolomics dataset with or without improper spectral alignment as the input samples. UV scaling was demonstrated to be a robust approach for extracting the clustering information of NMR metabolomics data. This conclusion was further confirmed by PCA of the NMR datasets generated from spleen tissue (mice), serum (mice), and cell (*Staphylococcus aureus*) samples. For discriminative metabolite identification purposes, scaling algorithm-dependent advantages and disadvantages were observed. The UV scaling method performed well in identifying the discriminative metabolites with their relative quantities significantly perturbed by an 800 m run. However, only when Par scaling and CTR scaling were applied could the discriminative metabolites with significant changes in their absolute quantities be easily identified. Specifically, the top two metabolites with the highest absolute quantity changes were lactate and creatinine. They are excise relevant, and their quantity changes observed in the experiments match well with the biological context.

## 2 Materials and methods

### 2.1 Selection and description of participants

Thirty-six athletes (age, 16.3 ± 1.8 yeras; body mass, 57.8 ± 4.3 kg; height, 175.3 ± 4.3 cm; body mass index, 18.8 ± 1.1 kg m^−2^; fat percentage, 10.7 ± 1.4%; maximum oxygen uptake, 66.0 ± 3.0 mL kg^−1^ min^−1^; all are mean values ± SEM) were recruited from Shanghai University of Sport and defined as group 1 (for dataset 1 and dataset 2). Twelve athletes (age, 16.3 ± 1.9 yeras; body mass, 56.7 ± 4.2 kg; height, 174.9 ± 5.1 cm, body mass index 18.5 ± 0.8 kg m^−2^; fat percentage, 10.2 ± 1.2%; maximum oxygen uptake, 66.0 ± 3.0 mL kg^−1^ min^−1^; all are mean values ± SEM) were selected from Shanghai University of Sport and defined as group 2 (for dataset 3 and dataset 4). During the experiments, athletes in group 2 took an 800 m run. All athletes were given a standard diet, water, and 3 days rest before urine sample collection or exercise (800 m run). This experimental setup is a common one for the participants who were not deliberately chosen, and also a customized and new one customized to this paper for the data will be intentionally adjusted with or without spectral peak alignment to display different visualization effects. The abovementioned parameters are not statistically significant between the two groups of athletes.


**Informed consent**: Informed consent has been obtained from all individuals included in this study.
**Ethical approval:** The research related to human use has been complied with all the relevant national regulations, institutional policies, and in accordance with the tenets of the Helsinki Declaration, and has been approved by the ethics committee of Shanghai University of Sport.

### 2.2 Sample collection and preparation

The procedures of urine sample collection and preparation were following the procedure described by Ma et al. [15]. Midstream urine samples of 36 athletes in group 1 were collected after lunch (at 2 pm) in solid CO_2_-cooled tubes (15 mL) containing 250 μL of 0.1% (w/v) sodium azide. The 12 athletes in group 2 took an 800 m run in approximately 1.93 min and then enjoyed a 15 min rest. Their midstream urine specimens were collected before and after exercises in solid CO_2_-cooled tubes (15 mL) containing 250 μL of 0.1% (w/v) sodium azide. Aliquots of urine (600 μL) obtained from each participant were centrifuged at 4,000 × *g* for 10 min at 4°C. The supernatant (550 μL) was mixed with 60 μL of D_2_O phosphate buffer (1.5 M KH_2_PO_4_, pH 7.4) containing 0.01% sodium 3-(trimethylsilyl) [2, 2, 3,3-D4] propionate (TSP) and then transferred into a 5 mm NMR tube for NMR analysis.

### 2.3 ^1^H NMR spectroscopy of urine

NMR experiments of urine samples were performed in a Bruker Avance III 600 MHz spectrometer (Karlsruhe, Germany) equipped with a cryoprobe at 300 K. Solvent-suppressed 1D ^1^H NOESY spectra (NoesyPr1d) with the pulse sequence [RD-90-t_1_-90-t_m_-90-ACQ] were run with four dummy scans and 64 free induction decays (FIDs) with a recycle delay (RD) of 10 s, a mixing time of 100 ms, and an acquisition time (ACQ) of 2.67 s. The 90° pulse length was adjusted to approximately 14.05 µs. The spectra were collected into 64k acquisition points, covering a spectral width of 12 kHz (20 ppm). Water resonance was suppressed by a pre-saturation pulse implemented during the RD and the mixing time.

### 2.4 Data reduction and technical variation

**Figure 1:**
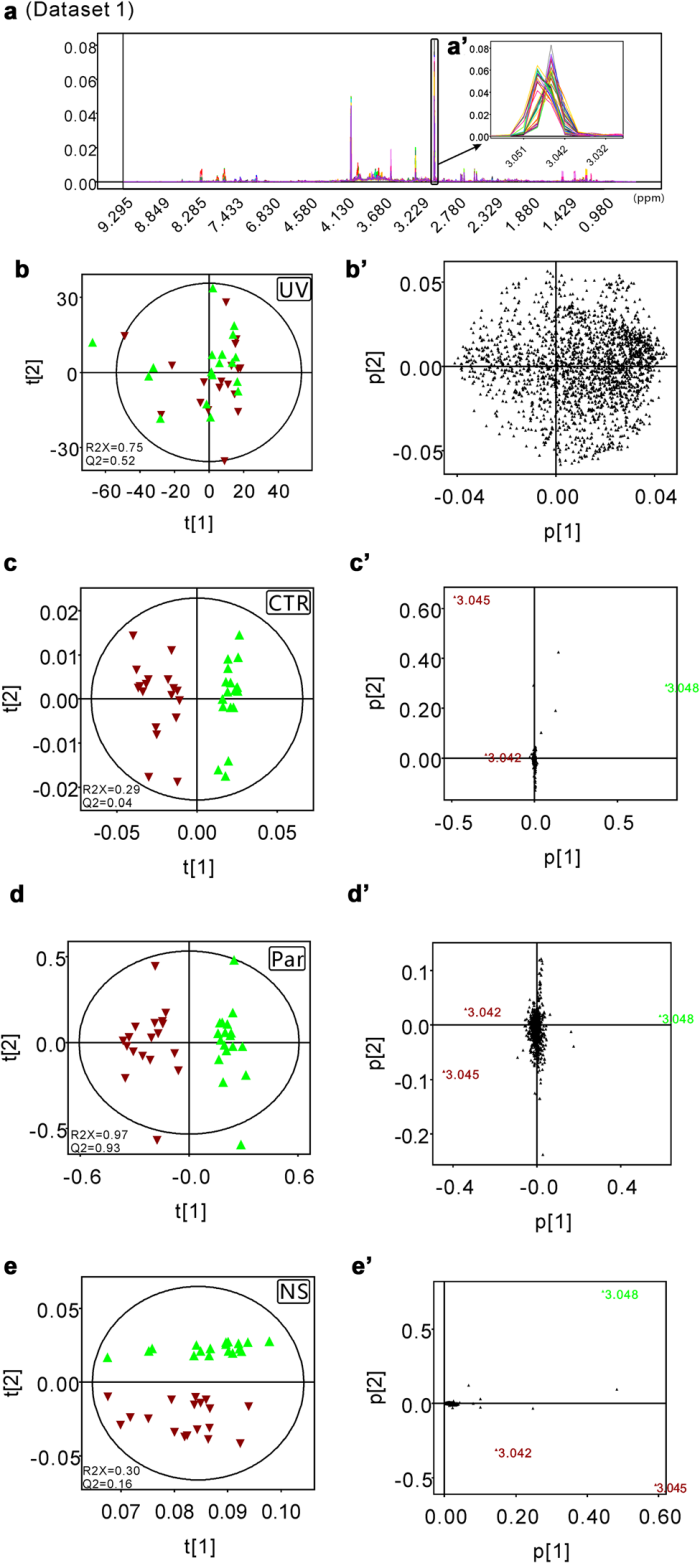
The scatter and loading plots obtained from the PCA models of dataset 1 generated from the 1D ^1^H-NMR spectra of the urine samples from 36 athletes. (a and a′) The processed spectral data with improper peak alignment in the region spanning from 3.042 to 3.051 ppm incorporated. (b and b′) The PCA scatter plot and the PCA loading plot generated from the UV scaling preprocessed dataset 1. (c and c′) The PCA scatter plot and the PCA loading plot generated from CTR scaling preprocessed dataset 1. (d and d′) The PCA scatter plot and the PCA loading plot generated from the Par scaling preprocessed dataset 1. (e and e′) The PCA scatter plot and the PCA loading plot generated from the NS preprocessed dataset 1.

**Figure 2:**
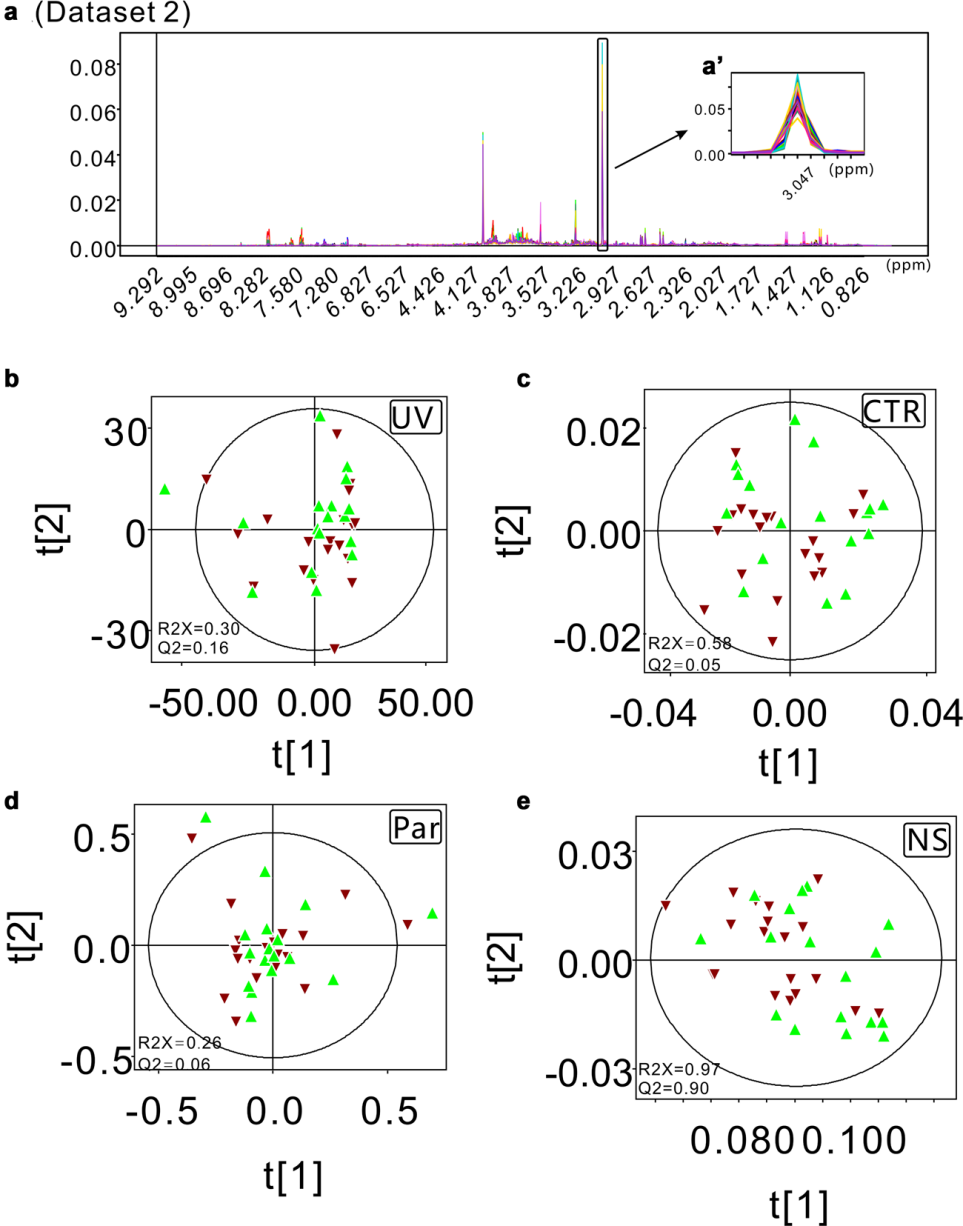
Scatter plots obtained from the PCA models of dataset 2 with the proper peak alignment spanning the full region of the 1D ^1^H-NMR spectra from the urine samples of 36 athletes. (a and a′) The processed spectra data with the proper peak alignment spanning the full region of the spectra are presented. (b) The PCA scatter plot generated from the UV scaling preprocessed dataset 2. (c) The PCA scatter plot generated from the CTR scaling preprocessed dataset 2. (d) The PCA scatter plot generated from the Par scaling preprocessed dataset 2. (e) The PCA scatter plot generated from the NS preprocessed dataset 2.

**Figure 3:**
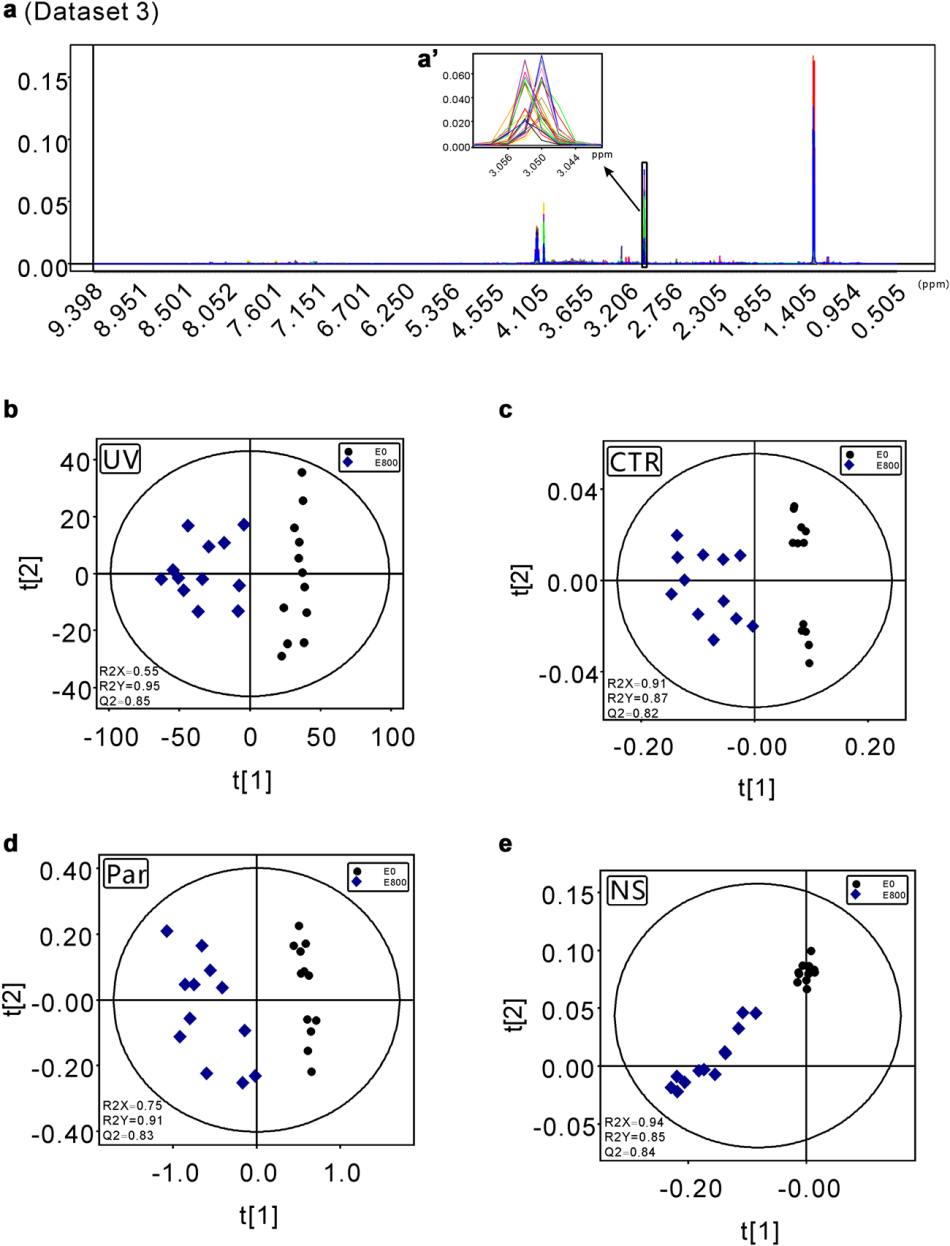
The scatter plots obtained from the PLS-DA models of dataset 3 generated from the 1D ^1^H-NMR spectra of the urine samples of 12 young athletes before (E0) and after exercises (E800). (a and a′) The processed spectral data with improper peak alignment in the region spanning from 3.044 to 3.051 ppm incorporated. (b) The PLS-DA scatter plot generated from the UV scaling preprocessed dataset 3. (c) The PLS-DA scatter plot generated from CTR scaling preprocessed dataset 3. (d) The PLS-DA scatter plot generated from the Par scaling preprocessed dataset 3. (e) The PLS-DA scatter plot generated from the NS preprocessed dataset 3.

**Figure 4:**
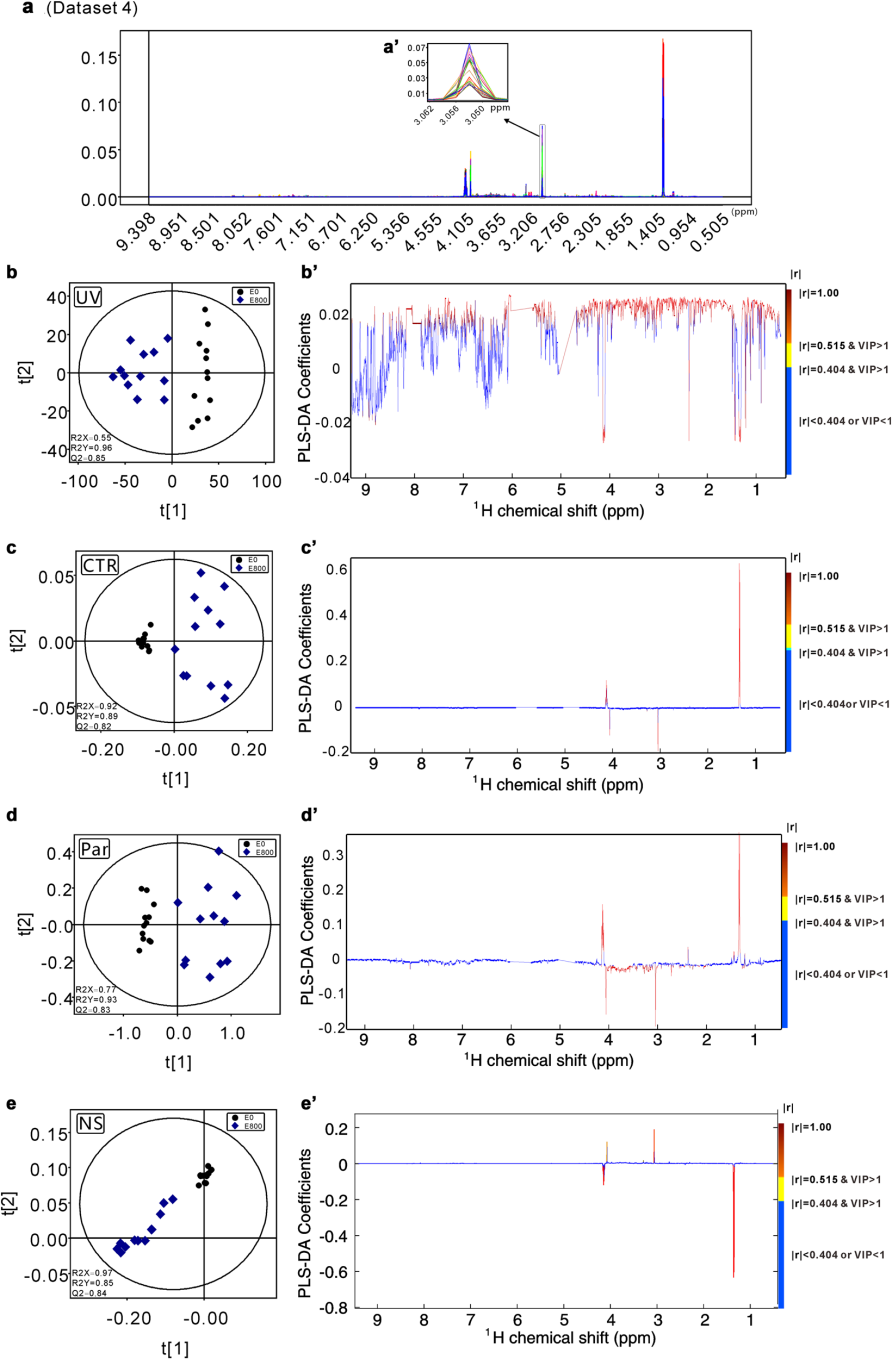
The scatter and loading plots obtained from the PLS-DA models of dataset 4 generated from the 1D ^1^H-NMR spectra of the urine samples of 12 young athletes before (E0) and after exercises (E800). (a and a′) The processed spectra data with the proper peak alignment spanning the full region of the spectra are presented. (b and b′) The PLS-DA scatter plot and the PLS-DA loading plot generated from the UV scaling preprocessed dataset 4. (c and c′) The PLS-DA scatter plot and the PLS-DA loading plot generated from the CTR scaling preprocessed dataset 4. (d and d′) The PLS-DA scatter plot and the PLS-DA loading plot generated from the Par scaling preprocessed dataset 4. (e and e′) The PLS-DA scatter plot and the PLS-DA loading plot generated from the NS preprocessed dataset 4. Metabolites with VIP values larger than 1 and correlation coefficient (*r*) values greater than 0.404 or less than −0.404 indicate that their levels are changed with statistical significance.

Raw data were processed following the procedure described by Liu et al. [22]. First, all the FIDs were multiplied by an exponential function of a 0.3 Hz line-broadening factor to increase the signal/noise ratio, and then all NMR spectra were manually phased and baseline corrected. After that, the peaks in the 1D NMR spectra of the 36 athletes’ urine samples were carefully aligned by using MestReNova software (Version 8.1.2, Mestrelab Research S.L.) except for the special peak position (chemical shift ranging from 3.042 to 3.051 ppm) of creatinine (dataset 1, Figure 1). On the other hand, the data with all peaks well aligned were assigned as dataset 2 (Figure 2). Then, all processed spectra were binned into 3,000 bins of width of 0.003 ppm corresponding to the chemical shift range of 0.50–9.50 ppm, and the residual water resonance (4.50–6.00 ppm) was excluded from these operations. The peaks in the 1D NMR spectra of the 12 athletes’ urine samples before and after exercise were carefully aligned except for the special peak position (chemical shift ranging from 3.044 to 3.056 ppm) of creatinine (dataset 3, Figure 3). The data with all peaks well aligned were assigned as dataset 4 (Figure 4). The signals of residual water (4.50–6.00 ppm) were not included in the processing operations. After all the data were normalized to the total sum of integrals, the matrix data were imported into SIMCA-P + software for statistical analysis (Version 12.0, Umetrics AB, Umeå, Sweden). All four datasets were processed in parallel by UV scaling, CTR scaling, Par scaling, and NS. After that, for datasets 1 and 2, unsupervised PCA was performed to detect the clustering tendency and the potential outliers within the samples. The loading plots were calculated to extract the most important variables for the discrimination between clusters. For datasets 3 dataset 4, PLS-DA was applied to obtain the cluster separation information. The results were visualized in the form of scatter plots to show the distribution of data points and loading plots to identify the discriminative buckets contributing to the cluster separation.

### 2.5 Autoscaling all variables to UV scaling

UV scaling, also called autoscaling, controls the distance between each data point by setting the standard deviation of all of the variables to one [20]. After being scaled by the UV approach, each of the variables in the dataset was modified by its respective UV weight, which confers equal importance to all the variables, including the signals and the noises. UV scaling is intended to compress the data amplitude variations and magnify those data with less variation, which enhances the contributions of the data derived from the noise signals in the spectra [23,24], and thus enlarges the tolerance to technical errors. However, the illustration effect on tolerance to technical errors of UV scaling on the clustering profiling have not yet been demonstrated in the previous literature.

### 2.6 CTR scaling

CTR scaling, which centers the variables by subtracting their averages, is the most commonly used approach in processing the spectra (MS/NMR) data. Since the scaling factor in the CTR scaling operation is different for each variable, the processed variables fluctuate around the zero level. In comparison with the UV scaling method, the statistical models derived from the dataset processed by the CTR scaling approach are more sensitive to those variables with larger values [25]. And thus, we will enlarge the variation in the top metabolite with high level. Different characteristics of tolerance to technical errors of CTR scaling compared with other scaling methods on the clustering discrimination will be demonstrated in the present work.

### 2.7 Par scaling

Par scaling first scales the variables by mean CTR and then divides each variable with the square root of its standard deviation [20]. In comparison with UV scaling and CTR scaling, Par scaling confers partially compromised weights to all of the variables. Par scaling intends to enhance the contributions of the data derived from the signals with smaller standard deviations. And the visualization features on the cluster profiling of Par scaling compared to the other scaling methods will be demonstrated in the work.

## 3 Results and discussion

In the present work, scatter plots of PCA were adopted to investigate the clustering tendencies of datasets 1 and 2 preprocessed with four different scaling methods. PCA loading plots were generated and applied to extract the most important variables for the discrimination between clusters. The results were visualized in the form of scatter plots to show the distribution of data points and loading plots to identify the discriminative buckets contributing to the cluster separation.

### 3.1 UV scaling showed larger technical error tolerance on NMR-derived metabolomics dataset than CTR scaling, Par scaling, and NS

The PCA scatter plots and loading plots derived from the dataset preprocessed with four different scaling algorithms showed significantly different visualization features. As described in Section 2, the buckets centered at 3.045 and 3.048 ppm in dataset 1 (Figure 1a′) were not aligned very well, while they were aligned correctly in dataset 2 (Figure 2a′). The UV scaling approach showed a larger tolerance to this technical error (poor signal alignment). The scatter plots from the two components based on the UV scaling processed data generated from NMR datasets 1 and 2 are strikingly similar to each other (Figures 1b and 2b). In fact, in the two PCA scatter plots derived from the UV scaling preprocessed datasets 1 and 2, the sample specimens are well clustered in the middle of the plots, and their distribution patterns in the two plots are almost the same (Figures 1b and 2b). In addition, the corresponding PCA parameters for datasets 1 and 2 also show no significant differences. Similar values of *R* ^*2*^ *X* and *Q* ^*2*^ related to the two models presented in Figure 1b (*R* ^*2*^ *X* = 0.30, *Q* ^*2*^ = 016) and Figure 2b (*R* ^*2*^ *X* = 0.30, *Q* ^*2*^ = 0.16) indicate that their explanation abilities are almost equal to each other. All the abovementioned data suggest that UV scaling might serve as a robust approach to the extraction of the clustering information of NMR metabolomics data, especially when technical errors exist. This conclusion is further confirmed by the statistical analysis results derived from datasets 3 and 4 (Figures 3 and 4). In the PLS-DA score plots generated from the UV scaling preprocessed datasets 3 and 4, clear group separations with similar scattering patterns were observed. Additionally, in the function of clustering profiling, there are demarcation lines either with wrong location in the scatter plots (Figures 3d and 4e) or abnormal direction between clusters (Figures 1e and 2e) with NS method. Because of the worst clustering performance, there is no need to investigate the function in the metabolite’s discrimination of NS method.

In comparison with the clustering analysis results obtained by preprocessing the datasets with the UV scaling method, the applications of the other three scaling approaches caused totally different consequences. The pairwise scattering patterns in the PCA scatter plots generated from the CTR scaling, Par scaling, and NS preprocessed datasets 1 and 2 are significantly different from each other (Figures 1 and 2). When the technical error is incorporated (dataset 1), the unwanted cluster separation tendencies were observed in the PCA data derived from the CTR scaling, Par scaling, and NS preprocessed dataset 1 (Figure 1). These results suggest that all three scaling methods are sensitive to technical errors from the NMR data. Consistent with the PCA scatter plot data, the data points derived from the misaligned buckets centered at 3.045 and 3.048 ppm bump out from the other data points in the loading plots generated from the CTR scaling, Par scaling, and NS preprocessed dataset 1, which indicate that they make significant contributions to the observed cluster separation tendencies in the PCA scatter plots (Figure 1). Different from the PCA results generated from the CTR scaling, Par scaling, and NS preprocessed dataset 1, no cluster separation tendencies were detected when dataset 2 was used to perform the PCA (Figure 2). We can conclude that the three scaling algorithms, CTR scaling, Par scaling, and NS, are all less robust than the UV method for the extraction of clustering information. However, with a skillful pretreatment of the raw metabolomics data, reliable and satisfactory statistical results can be achieved. The abovementioned conclusion was further confirmed by PCA of the NMR datasets generated from spleen tissue (mice), serum (mice), and cell (*Staphylococcus aureus*) samples (please see supplementary materials for details).

### 3.2 Specific signatures were observed for UV scaling, CTR scaling, and Par scaling in identifying the discriminative metabolites between groups

Identifying the discriminative metabolites that contribute significantly to the group separations is the key step in the metabolomics analysis. The cutoff value of *r* (correlation coefficient) with a significance level of 0.05 and the variable importance in projection (VIP) value larger than 1 are the major standards to define the variables (metabolites) that are most correlated to the detected group separations [26,27]. To investigate the performances of four scaling methods in identifying the discriminative metabolites, PLS-DA analysis for UV scaling, CTR scaling, Par scaling, and NS preprocessed dataset 4 was carried out. The obtained data demonstrated that in comparison with UV scaling, the other three methods are more user-friendly for discriminative metabolite identification purposes. In the PCA loading plots generated from the CTR scaling, Par scaling, and NS preprocessed NMR datasets, the discriminative metabolites with their signals colored yellow or red could be easily read out (Figure 4). However, when the UV scaling method was used, only the skillful expertise could unambiguously select the discriminative metabolites from the noises (Figure 4). However, when the CTR scaling approach was applied, significant data inconsistencies between the pairwise correlation coefficient and the VIP value of the identified discriminative metabolites were observed in the loading plot-derived list (Table 1). When the Par scaling method was used, less severe inconsistencies were detected (Table 1). Overall, when both the correlation coefficient value (*r*) and the VIP value were considered to pick out the discriminative metabolites, in comparison with the UV scaling application (a total of 21 discriminative metabolites were identified), fewer discriminative metabolites were identified when CTR (only 3 discriminative metabolites were identified) or PAR scaling (a total of 14 discriminative metabolites were identified) were used. All the biological processes are quite complicated, and their unique metabolic signatures can only be defined by a systematic metabolic network instead of a limited number of perturbed metabolites. Therefore, the CTR scaling algorithm did a worse job since only three discriminative metabolites, including lactate, creatinine, and π-methylhistidine, were prejudicially screened out (Table 1). However, the VIP values of identified discriminative metabolites generated from the NMR datasets preprocessed by using the CTR or the PAR scaling method match well with the absolute changes in the quantity of metabolites (Table 1), which indicates that the discriminative metabolites with significant changes in their absolute quantities could be more easily identified by using these two scaling algorithms. Specifically, the top two metabolites with the highest absolute quantity changes were lactate and creatinine. They are excise relevant, and their quantity changes observed in the experiments match well with the biological context. On the other hand, it is worth noting that the relative changes (percentage changes) in the quantity of metabolites, which are generally used to distinguish the most perturbed metabolites, are not VIP value relevant (Table 1). This means that the UV scaling application could more efficiently identify discriminative metabolites with significant relative quantity changes. In the process of comparing Par and CTR scaling (Table 1), if only correlation coefficient (*r*) value was used to identify the discriminative metabolites between clusters, the results from CRT and Par are similar. If *r* and VIP values are used together to identify distinct metabolites, the results from Par scaling method will be better than the ones from CTR scaling methods, thus Par scaling will be chosen as the better one in their pipeline.

**Table 1:** Identification of discriminative metabolites between groups (E0 and E800) extracted from the PLS-DA loading plot with three different scaling methods (UV, CTR, and Par)

Metabolites (ppm) (HMDB ID)	Relative quantity changes (%) (P*)	UV (*r* ^#^, VIP^$^)	CTR (*r* ^#^, VIP^$^)	Par (*r* ^#^, VIP^$^)
2-Aminoadipate (2.29) (HMDB00510)	−48.3 (0.00)	0.74, 1.01	0.74, 0.09	0.74, 0.86
2-Hydroxybutyrate (0.89) (HMDB00008)	−33.5 (0.00)	0.89, 1.30	0.87, 0.04	0.88, 0.69
3-Aminoisobutyrate (1.18) (HMDB03911)	−55.5 (0.00)	0.90, 1.36	0.89, 0.05	0.91, 0.77
3-Hydroxyisovalerate (1.28) (HMDB00754)	−45.9 (0.00)	0.88, 1.26	0.84, 0.07	0.88, 0.85
Alanine (1.50) (HMDB00161)	−31.1 (0.00)	0.84, 1.03	0.82, 0.02	0.81, 0.43
Choline (3.19) (HMDB00097)	−63.2 (0.00)	0.91, 1.42	0.90, 0.11	0.93, 1.02
Citrate (2.56) (HMDB00094)	−57.0 (0.00)	0.90, 1.28	0.88, 0.10	0.90, 1.02
Creatine (3.96) (HMDB00064)	−55.8 (0.00)	0.88, 1.30	0.92, 0.42	0.93, 2.04
Creatinine (3.05) (HMDB00562)	−50.0 (0.00)	0.89, 1.26	0.90, 9.91	0.91, 10.0
Dimethylamine (2.72) (HMDB00087)	−53.8 (0.00)	0.90, 1.32	0.88, 0.04	0.94, 2.46
Glycine (3.57) (HMDB00123)	−49.8 (0.00)	0.83, 1.20	0.81, 0.36	0.84, 1.87
Inosine (6.08) (HMDB00195)	−60.7 (0.00)	0.93, 1.42	0.97, 0.01	0.97, 0.38
Isobutyrate (1.08) (HMDB01873)	−25.9 (0.00)	0.82, 1.23	0.80, 0.01	0.81, 0.41
Lactate (1.35) (HMDB00190)	800.5 (0.00)	0.97, 1.38	0.99, 32.9	0.97, 19.1
*N*,*N*-Dimethylglycine (2.93) (HMDB00092)	−62.9 (0.00)	0.79, 1.24	0.79, 0.19	0.79, 1.40
*N*-Acetylcysteine (2.07) (HMDB01890)	−42.8 (0.00)	0.91, 1.25	0.90, 0.11	0.92, 1.07
*N*-Acetylglutamate (2.05) (HMDB01138)	−39.5 (0.00)	0.95, 1.29	0.94, 0.11	0.96, 1.08
Succinate (2.38) (HMDB00254)	211.6 (0.00)	0.97, 1.36	0.98, 0.33	0.97, 1.91
Taurine (3.43) (HMDB00251)	−59.3 (0.00)	0.84, 1.20	0.75, 0.32	0.76, 1.70
Trimethylamine N-oxide (3.28) (HMDB00925)	−55.0 (0.00)	0.86, 1.26	0.53, 0.90	0.51, 2.11
π-Methylhistidine (3.69) (HMDB00479)	−42.9 (0.00)	0.88, 1.28	0.80, 1.38	0.82, 1.90

*P, the statistical significance value obtained from the pairwise comparisons of Student s t test.

^#^The correlation coefficient value (*r*) obtained from E0 vs. E800 models which is greater than 0.40 suggest that the concentration of the metabolite with this value has statistical difference between groups.

^$^The VIP value which is greater than 1 suggests that this variable has difference between groups.

Overall, our data indicate that although not user-friendly, UV scaling is more robust for the selection of discriminative metabolites with significant relative quantity changes in metabolomics analysis based on NMR-derived spectral data. However, to paint a more precise picture of the discriminative metabolites, the Par scaling and CTR scaling methods should be jointly applied.

## 4 Conclusion

In this work, the performances of three scaling approaches (UV, CTR, and Par) in NMR-based metabolomics analysis were systematically investigated, and the robustness and limitations of UV scaling, CTR scaling, and Par scaling were revealed. In our study, UV scaling gave robust results in both the extraction of clustering information and the identification of discriminative metabolites. In comparison with CTR scaling and Par scaling, UV scaling is much less insensitive to the technical errors incorporated in the analysis (Figures 1 and 2). To achieve optimal performance in NMR-based metabolomics analysis, UV scaling, Par scaling, and CTR scaling should be jointly used. In the clustering tendency detection step, the UV scaling approach is recommended. After the clustering information is obtained, the CTR scaling and Par scaling methods could be applied to detect possible technical error incorporated into the analysis. Then, with the guidance of the results revealed by the PCA scatter plot and loading plot derived from the CTR scaling or Par scaling preprocessed NMR spectra data, the technical errors can be fixed. After this operation, the discriminative metabolites contributing to group separations will be identified efficiently by using the loading plot data obtained in the new cycle of statistical analysis of the UV scaling. Overall, our data demonstrate an optimal working pipeline for scaling algorithm selection in NMR-based metabolomics analysis.

## Supplementary Material

Supplementary material
