# Supplementary material for: Performance comparison of three scaling algorithms in NMR-based metabolomics analysis
Source: Open Life Sci. 2023 Mar 27;18(1):20220556. doi: 10.1515/biol-2022-0556 (PMC10044292; doi:10.1515/biol-2022-0556)
Supplement: Supplementary material [file biol-2022-0556-sm.pdf]

Supplementary material

S1 Supplementary materials and methods

S1.1 Sample collection

To test the conclusion presented in the manuscript, NMR datasets from tissue, serum, and cell samples were collected to investigate the sample scattering profiles when processing the datasets with different scaling methods. Spleen tissue samples were harvested from 18-month-old APP/PS1 double transgenic female mice (APP/PS1, n = 6) and 18-month-old wild-type (B6C3F1) female mice (WT, n = 6). The hydrophilic metabolites extracted from the spleen samples were processed by using an extraction system of CH_3OH, $CHCl_3$ and H_2O and a tissue homogenizer (Precellys 24, Bertin Technologies, Villeurbanne, France). The extracts were lyophilized and redissolved in 550 µL of phosphate buffer (0.2 M Na_2HPO_4/0.2 M NaH_2PO_4/D_2O, pH 7.4). After centrifugation (11,000 rpm) at 4°C for 10 minutes, aliquots of the supernatants (500 L) were transferred into 5-mm NMR tubes for NMR data acquisition.

Serum samples were collected from 3-month-old male Sprague–Dawley rats treated with or without 28-O-caffeoyl botulin (WCB, n = 9, and control group, **CG**, n = 9). In addition, 300 µL phosphate buffer (0.2 M Na_2HPO_4/ 0.2 M NaH_2PO_4, pH 7.4) was then added to each plasma (300 µL) sample. After centrifugation (11,000 rpm) at 4°C for 10 minutes, the supernatants (500 µL) were moved into 5 mm NMR tubes containing 50 µL D2O with 0.01% sodium 3-(trimethylsilyl) [2, 2, 3,3-D4] propionate (TSP).

The *Staphylococcus aureus* specimens were cultured by using medium with or without glucose added. Overnight cultured *Staphylococcus aureus specimens* were transferred into TSB medium in the absence or presence of 0.25% glucose (AG, n=7, PG, n=7). Fifteen milliliters of fresh cultures was sampled after incubation for 12 hours, and the cell pellets were collected by centrifugation (4,500 g, 10 min) at 4°C. Cells were washed twice with 15 mL of precooled PBS buffer and resuspended in phosphate buffer (1 mL, 50 mM, dissolved in D_2O). After being disrupted by a cell disrupter (Fast Prep FP, 6.5 M/S, 40 S, 3 times), cell debris was removed by centrifugation at 15,000 g and 4°C for 10 minutes. Aliquots of the supernatants (600 µL) were transferred into 5 mm NMR tubes for NMR data acquisition.

S1.2 ^{1}H NMR spectroscopy

Data were acquired in a random, blinded order on a Bruker (Karlsruhe, Germany) Avance III 600 MHz NMR spectrometer equipped with a cryoprobe operating at 600.13 MHz and 300 K. For the NMR data of spleen samples, a solvent-suppressed 1D ^{1}H ZGPR pulse sequence (RD-90°-ACQ) was used to record the ^{1}H NMR spectra with four dummy scans and 128 transients into 32 K data points using a spectral width of 20 ppm with a elaxation delay of 10.0 seconds and an acquisition time of 2.73 seconds.

The NMR spectra of serum and cell samples were acquired using a 1D technique using a standard pulse sequence with presaturation of the water resonance and Carr–Purcell–Meiboom–Gill (CPMG) spin echo sequences to attenuate the broader peaks arising from lipids and proteins. Samples were calibrated and referenced using the sodium 3-(trimethylsilyl) [2, 2, 3,3-D4] propionate peak at 0.00 ppm. For each sample, 256 transients (FIDs) were acquired into 32 K complex data points over a spectral width of 20 ppm.

S1.3 Data processing for statistical analyses

FID processing was conducted by using the software MestReNova Version 8.1.4 (Mestrelab Research S.L.), and Fourier transformation was performed with an exponential weighting factor of 0.3 Hz line-broadening factor to improve the signal-to-noise ratio of the spectra. Spectra were phased, corrected for baseline distortion, and carefully aligned (Figure S1A, Figure S2A, Figure S3A) or improperly aligned (misaligned at 3.224–3.227 ppm in Figure S1B, misaligned at 1.340–1.343 ppm in Figure S2B, misaligned at 3.043–3.049 ppm in Figure S3B). Then, the spectral region of δ 9.50–0.50 was segmented into 3000 bins with a 0.003 ppm width for each bin. The bins were

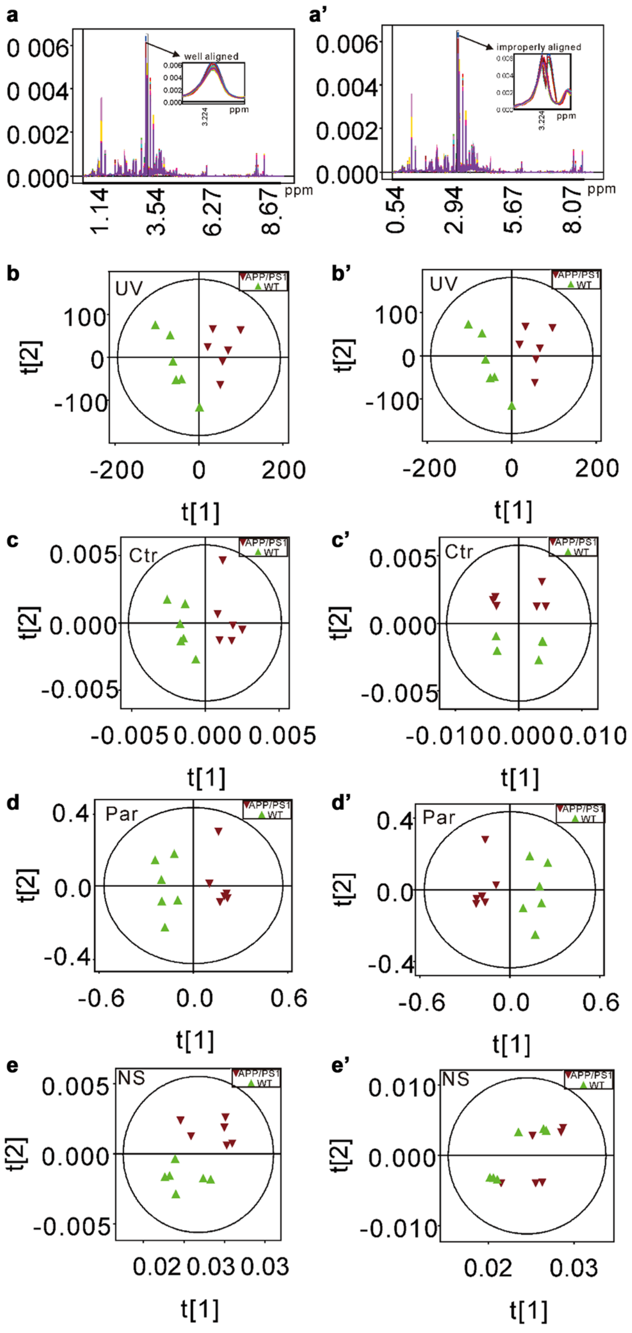

Figure S1: Scatter plots obtained from the PCA models of the datasets generated from the spleen tissue samples. (a, a') The processed spectra data without or with the improper peak alignment in the region spanning from 3.224 ppm to 3.228 ppm incorporated. (b, b') The PCA scatter plots generated from the UV scaling preprocessed NMR datasets either without (b) or with (b') the improper peak alignment incorporated. (c, c') The PCA scatter plots generated from the CTR scaling preprocessed NMR datasets either without (c) or with (c') the improper peak alignment incorporated. (d, d') The PCA scatter plots generated from the Par scaling preprocessed NMR datasets either without (d) or with (d') the improper peak alignment incorporated. (e, e') The PCA scatter plots generated from the raw NMR datasets (no scaling approach was applied) either without (e) or with (e') the improper peak alignment incorporated.

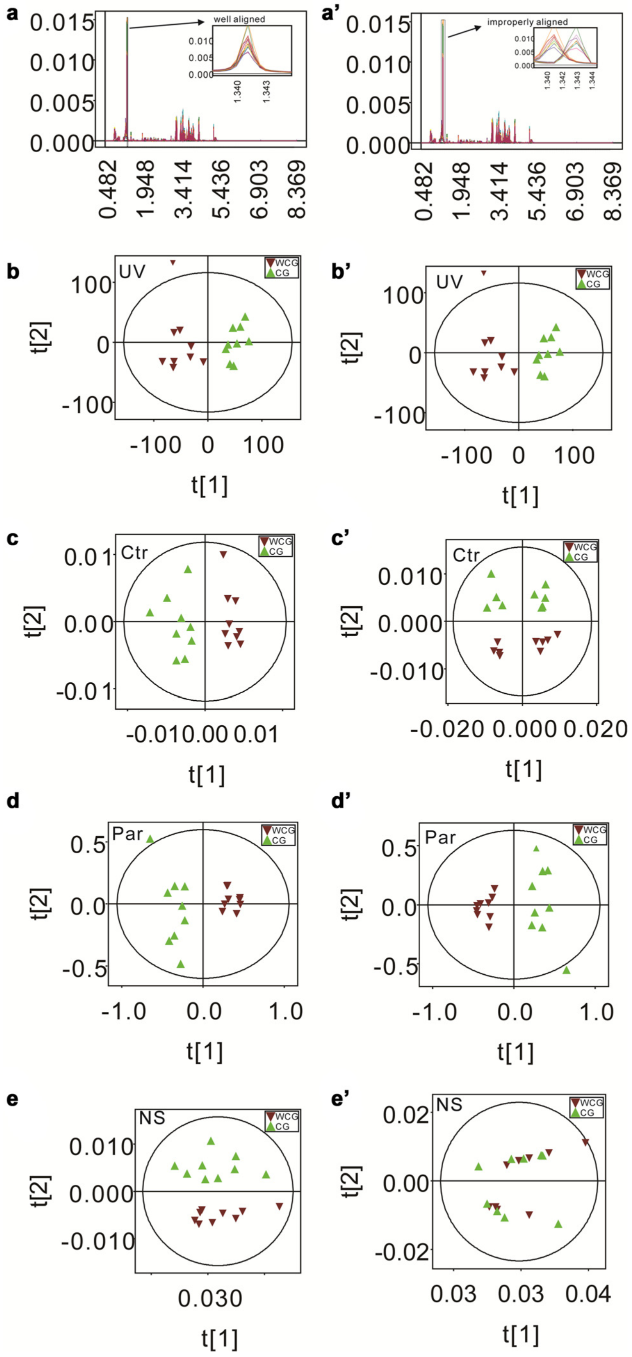

Figure S2: Scatter plots obtained from the PCA models of the datasets generated from the serum samples of rats with or without 28-O-caffeoyl botulin treatment. (a, a') The processed spectra data without or with the improper peak alignment in the region spanning from 3.224 ppm to 3.228 ppm incorporated. (b, b') The PCA scatter plots generated from the UV scaling preprocessed NMR datasets either without (b) or with (b') the improper peak alignment incorporated. (c, c') The PCA scatter plots generated from the CTR scaling preprocessed NMR datasets either without (c) or with (c') the improper peak alignment incorporated. (d, d') The PCA scatter plots generated from the Par scaling preprocessed NMR datasets either without (d) or with (d') the improper peak alignment incorporated. (e, e') The PCA scatter plots generated from the raw NMR datasets (no scaling approach was applied) either without (e) or with (e') the improper peak alignment incorporated.

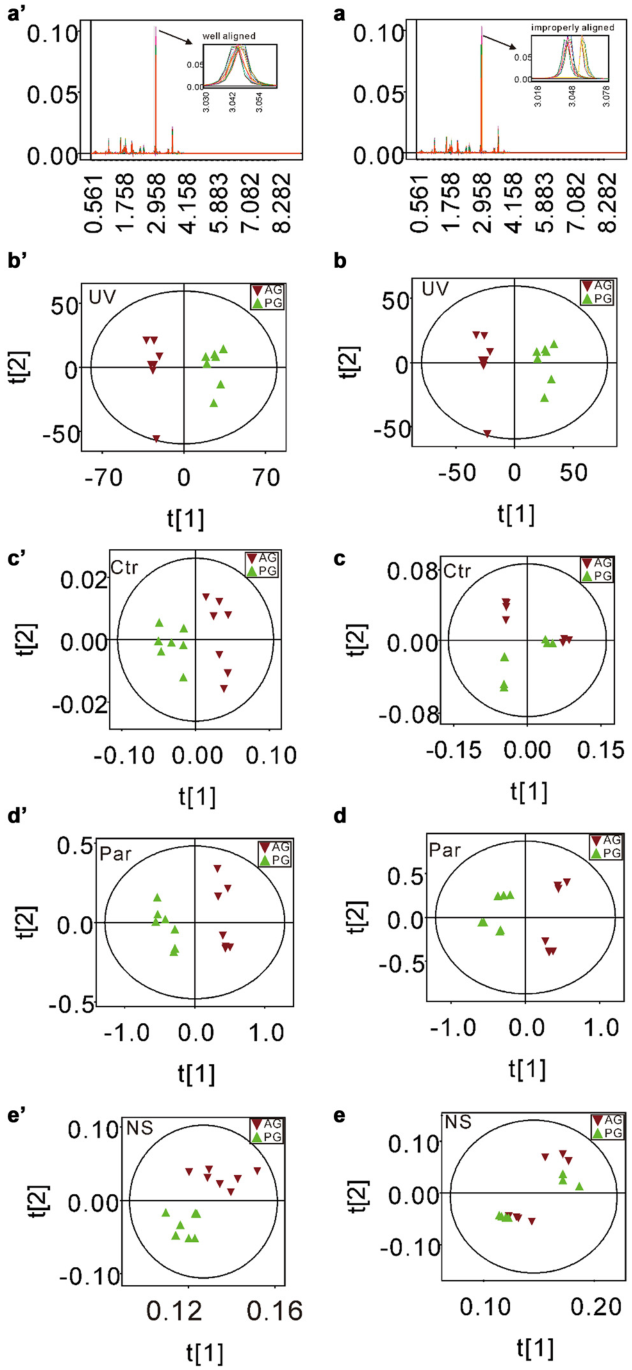

Figure S3: Scatter plots obtained from the PCA models of the datasets generated from the cell samples of *Staphylococcus aureus* cultured in medium without or with 0.25% glucose added. (a, a') The processed spectra data without or with the improper peak alignment in the region spanning from 3.224 ppm to 3.228 ppm incorporated. (b, b') The PCA scatter plots generated from the UV scaling preprocessed NMR datasets either without (b) or with (b') the improper peak alignment incorporated. (c, c') The PCA scatter plots generated from the CTR scaling preprocessed NMR datasets either without (c) or with (c') the improper peak alignment incorporated. (d, d') The PCA scatter plots generated from the Par scaling preprocessed NMR datasets either without (d) or with (d') the improper peak alignment incorporated. (e, e') The PCA scatter plots generated from the raw NMR datasets (no scaling approach was applied) either without (e) or with (e') the improper peak alignment incorporated.

labeled with their median chemical shift value. The integrals from the region of δ 6.50–4.60 were excluded from the analysis to eliminate the effects of the distorted baseline from imperfect water saturation. The integrals of the remaining bins were normalized to 1 and used as a dataset in the following multivariate analysis. The processed data were imported into SIMCA-P software (Version 14.0, Umetrics AB, Umea, Sweden) for multivariate pattern recognition analyses. Principal component analyses (PCA) with UV scaling, CTR scaling, Par scaling, and no scaling (NS) were performed to detect group separation.

S1.4 Supplementary results and discussion

UV scaling gave robust clustering information for all of the datasets (Figure S1B vs Figure S1 B', Figure S2B vs Figure S2B', Figure S3B vs Figure S3B'). In comparison with CTR scaling, Par scaling, and no scaling, UV scaling is much less insensitive to the technical errors incorporated in NMR-based metabolomics analysis. In conclusion, UV scaling is suggested to be applied in the preliminary analysis of NMR-based metabolomics data, while CTR scaling should be carried out when technical errors such as improper peak alignment have been eliminated in NMR-derived datasets.
